# Neuro-Anatomical Evidence Indicating Indirect Modulation of Macrophages by Vagal Efferents in the Intestine but Not in the Spleen

**DOI:** 10.1371/journal.pone.0087785

**Published:** 2014-01-29

**Authors:** Cathy Cailotto, Pedro J. Gomez-Pinilla, Léa M. Costes, Jan van der Vliet, Martina Di Giovangiulio, Andrea Némethova, Gianluca Matteoli, Guy E. Boeckxstaens

**Affiliations:** 1 Tytgat Institute for Liver and Intestinal Research, Academic Medical Center (AMC), Amsterdam, The Netherlands; 2 Department of Gastroenterology, University Hospital Leuven, Catholic University of Leuven, Leuven, Belgium; Charité, Campus Benjamin Franklin, Germany

## Abstract

**Background:**

Electrical stimulation of the vagus nerve suppresses intestinal inflammation and normalizes gut motility in a mouse model of postoperative ileus. The exact anatomical interaction between the vagus nerve and the intestinal immune system remains however a matter of debate. In the present study, we provide additional evidence on the direct and indirect vagal innervation of the spleen and analyzed the anatomical evidence for neuroimmune modulation of macrophages by vagal preganglionic and enteric postganglionic nerve fibers within the intestine.

**Methods:**

Dextran conjugates were used to label vagal preganglionic (motor) fibers projecting to the small intestine and spleen. Moreover, identification of the neurochemical phenotype of the vagal efferent fibers and enteric neurons was performed by immunofluorescent labeling. F4/80 antibody was used to label resident macrophages.

**Results:**

Our anterograde tracing experiments did not reveal dextran-labeled vagal fibers or terminals in the mesenteric ganglion or spleen. Vagal efferent fibers were confined within the myenteric plexus region of the small intestine and mainly endings around nNOS, VIP and ChAT positive enteric neurons. nNOS, VIP and ChAT positive fibers were found in close proximity of intestinal resident macrophages carrying α7 nicotinic receptors. Of note, VIP receptors were found on resident macrophages located in close proximity of VIP positive nerve fibers.

**Conclusion:**

In the present study, we show that the vagus nerve does not directly interact with resident macrophages in the gut or spleen. Instead, the vagus nerve preferentially interacts with nNOS, VIP and ChAT enteric neurons located within the gut muscularis with nerve endings in close proximity of the resident macrophages.

## Introduction

In the last decade it has become clear that the vagus nerve fulfills an important role in modulating the immune system [1], [2]. Vagus nerve activation indeed has anti-inflammatory properties in a wide variety of disorders including systemic and local inflammation [3]–[8]. The first experiments leading to the introduction of this concept were performed in a rat model of sepsis [1], illustrating increased survival after vagus nerve stimulation. This effect is now believed to result from vagal activation of sympathetic neurons located in the mesenteric ganglion [9] rather than a direct effect of the vagus nerve in the spleen. These adrenergic nerve fibers release noradrenalin activating splenic T cells. These T cells subsequently release acetylcholine (Ach) that inhibits the release of pro-inflammatory cytokines from splenic macrophages through interaction with α7 (alpha7) nicotinic receptors [10], [11].

Also in the gastrointestinal tract, vagus nerve stimulation dampens the inflammatory response in several immune-mediated disorders, including postoperative ileus (POI). In the latter, intestinal manipulation initiates an inflammatory cascade through the activation of muscularis resident macrophages that results in delayed gastrointestinal motility. Electrical stimulation of the vagus nerve (VNS) and systemic administration of selective nicotinic receptor agonists dampened pro-inflammatory cytokine production by macrophages resulting in reduced intestinal inflammation and shortened POI [12]. Recently, we showed that this subtle inflammatory response evoked by manipulation of the small intestine elicits neuronal activation in the nucleus of the tractus solitarius (NTS) and the motor nucleus of the vagus nerve [13]. This vagal output targeted mainly the inflamed zone (intestine) but also other organs such as the spleen.

Although the innervation of the intestinal myenteric plexus by vagal efferents is well described [14], its interaction with the immune cells residing in the intestine is poorly characterized. Similarly, the innervation of the spleen is still a matter of controversy with some studies providing evidence of cholinergic innervation whereas others propose that the spleen is only innervated by sympathetic neurons located in the mesenteric ganglion [9], [13], [15]–[17]. Hence, we aim to provide neuro-anatomical evidence on the interaction between vagal efferents and resident macrophages in the intestine and to bring more clarity on the vagal innervation of the spleen in mouse. To this end we labeled the vagal motor efferent fibers arising from the dorsal motor nucleus (DMV) by using the dextran amines anterograde tracer, recently reported to provide high-definition labeling of vagal motor fibers [18].

## Materials and Methods

### Ethics Statement

All procedures were conducted in accordance with the Institutional guidelines and approved by the Animal Ethical Committee of the AMC/University of Amsterdam (reference protocol number 100096) and by the Ethical committee of the Catholic University of Leuven (Permit Number: 112/2011). All surgery was performed under anaesthesia (Hypnorm/Dormicum) and all effort was made to minimize suffering.

### Animals

Mice (female BALB/c; Harlan Nederland, Horst, The Netherlands) were kept in 12 h light/12 h dark cycle (lights on at 8:00 AM to 8:00 PM) under constant conditions of temperature (20±2°C) and humidity (55% humidity) with water and food *ad libitum*. Mice underwent surgical procedure at 11–13 weeks of age. Mice were anesthetized by FFM intraperitoneal injection, a mixture of fentanylcitrate/fluanisone (Hypnorm; Janssen, Beerse, Belgium) and midozolam (Dormicum; Roche, Mijdrecht, The Netherlands) in a ratio 1∶1∶2 (Hypnorm: Dormicum: H_2_0).

### Tracer injection

Mice were anesthetized and mounted on the stereotaxic frame. Bilateral injections of the biotin or Texas red dextran amines (5% solution, D-B, D-1956 or D-TRD1863, Invitrogen) were performed at different rostro-caudal levels of the DMV: AP −7.8 mm, −7.9 mm and −8.0 mm. The stereotaxic coordinates used for lateral ventricle injection were AP −0.5 mm, L −1.0 mm and V −2.0/−1.50/−1.0 mm. Injections (4 ms duration) were performed using a glass micropipette (25 µm). At the end of the injection procedure, the wound was closed by a suture with Mersilene, 6–0 silk.

### Tissue preparation

Nineteen days after injection of the tracer, anesthetized mice were sacrificed by transcardiac perfusion with Phosphate buffered saline (PBS) followed by 4% paraformaldehyde (PFA) (pH 7.4, 4°C). The spleen was removed prior the perfusion with fixative, quickly snap frozen and stored at −80°C. Then, brain, nodose and mesenteric ganglia and deep cervical lymph node were collected, post-fixed for 4 hrs (4°C) and immersed in 30% sucrose/0.2 M PBS (pH 7.4) overnight at 4°C. For the whole mount intestinal tissue preparation, samples were cut along the mesentery border, washed in cold saline and transferred to PFA for 4 hrs and to 30% sucrose. Prior to the immunohistochemical procedures, the muscle layers were gently stripped out from the mucosa and sub-mucosa with fine-tip forceps. For intestinal coronal sections, tissue were frozen in OCT embedding compound (Neg 50, Thermo Scientific, Walldrof, Germany) and stored at −80°C.

In some cases, colchicine was used to enhance VIP immunoreactivity of cell bodies. Intestinal tissues were washed with PBS containing gentamicine (1∶100 diluted) and incubated in Dulbecco's modified Eagle medium (Gibco, Life Technology) containing colchicine (0.01 g/100 ml) at 37°C. Following incubation, the tissue was stretched and fixed with Zamboni fixative.

### Immunohistochemical staining

#### Brainstem, nodose and mesenteric ganglia and deep cervical lymph node

Coronal sections of 30 µm for brainstem, 16 µm for nodose/mesenteric ganglia and deep cervical lymph nodes were collected. To reveal the biotin dextran amines, sections were pretreated first with a solution of Methanol (10%) and hydrogen peroxide H_2_O_2_ (0.1%) for 10 min and were subsequently incubated for 1 hour with avidin-biotin complex (ABC, Vector Labs PK-4000). The reaction product was visualized by incubation with 1% diaminobenzidine (DAB), 0.05% nickel ammonium sulfate and 0.01% H_2_O_2_ for 5 min.

To reveal the Texas red dextran amines, section was incubated overnight with the anti Texas red antibody (1/50; Invitrogen A6399) followed by an incubation overnight at 4°C with Goat anti-rabbit^Poly AP^ (1∶50; BrightVision Immunologic B.V, DPVR55AP). The reaction product was visualized by incubation with Alkaline Phosphatase Substrate Kit III (Vector Laboratories, Inc. SK-5300) for 20 minutes.

#### Intestinal whole mount preparation

ABC/DAB staining and the pan-neuronal marker cuprolinic blue were used to label dextran-labeled vagal fibers and enteric neurons, respectively. Briefly, the whole mount preparation was pretreated (30 min) with a solution Methanol- H_2_O_2_ (4∶1). The tissue was incubated for 4 hrs at 37°C with a solution of 0.5% cuprolinic blue (17052; Polyscience, Inc.) followed by incubation in buffer (0.05 M NaAC, 1 M MgCl_2_, pH 4.9) for 30 s. After thorough rinsing with distilled water and TBS, sections were incubated with ABC (1 hrs) and with 1% DAB 0.05% nickel ammonium sulfate and 0.01% H_2_O_2_ for 8 min. To visualize resident macrophages, preparations were exposed to the primary F4/80 antibody (1∶500; Biolegend, biotinylated rat antibody; 1∶200; Dako E0468) and were revealed by ABC/Nova Red (vector Labs, SK-4800).

### Immunofluorescent labeling

#### Coronal section of intestinal tissue

Sections underwent a treatment with Biotin-Blocking System from DAKO (protocol provided by the manufacturer). An additional blocking step was performed by two hours incubation with 1% bovine serum albumin (BSA, Sigma-Aldrich, St. Louis, MO) at room temperature (RT). Then, sections were treated with streptavidin conjugated with CY3 (1∶400; Jackson ImmunoResearch, diluted in 1% BSA+0.3% Triton X-100) for 1 hr at RT. A counterstaining with DAPI to label nuclei was used to delineate the anatomical structure of the intestine wall.

#### Intestinal whole mount preparation

The preparations were subjected to Biotin-blocking system (DAKO) and two hours incubation with 1% bovine serum albumin (BSA, Sigma-Aldrich, St. Louis, MO) at room temperature (RT). After blocking, the preparations were incubated with the primary antibodies (overnight, 4°C) rabbit anti-PGP 9.5 (1∶600,Chemicon), goat anti-ChAT (1∶5000, Chemicon) and rat anti-F4/80 (1∶200, Biolegend), rabbit anti-VIP (1∶2000, kindly provided by Prof. Dries Kalsbeek from the Netherlands Institute for Neuroscience, Amsterdam), rabbit anti-nNOS (1∶500, Santa Cruz), anti-PGP 9.5 (1∶500, Chemicon) and goat anti-VPAC1 (1∶500, Santa Cruz) diluted in PBS containing 1% BSA and 0.3% Triton X-100. Specificity of the primary antibodies used was confirmed by preincubation with the respective blocking peptide.

The next day, the tissues were incubated for 1 hour at room temperature with the following secondary antibodies at a concentration of 1∶1000; goat anti-rabbit CY3 conjugated (Jackson ImmunoResearch), donkey anti-goat FITC conjugated (Jackson ImmunoResearch), donkey anti-rat CY5 conjugated (Jackson ImmunoResearch), donkey anti-rabbit Alexa 555 (Molecular probe) or goat anti-rabbit alexa 555 (Molecular probe) and streptavidin conjugated with CY3 or CY5 (Jackson ImmunoResearch). The specificity of the secondary antibodies used or fluorescent streptovidin was confirmed by the lack of staining in the absence of preincubation with the primary antibody.

In case of nNOS/VIP, immunolabeling was performed sequentially including a step with citrate buffer prior to VIP labeling to avoid cross reactivity. In brief, the preparations were rinsed in citrate buffer (pH = 6) followed by 3 heating sessions (microwave 6 min at 600 W) and incubated in refresh citrate buffer for 20 min.

For α7 nicotinic receptor labeling [19] and macrophage staining (F4/80), jejunum tissue was incubated with FITC-labelled α-bungarotoxin (Invitrogen) at 0.1 µg/ml in RPMI 1640 medium (Lonza) at 4°C for 15 min. After thorough washes with PBS and post fixation with 4% of cold Paraformaldehyde (10 min), the mucosa and submucosa were gently removed from the muscle layers. The latter were subsequently processed for intestinal resident macrophages (F4/80) labeling.

#### Coronal section of spleen tissue

Eight µm sections were labeled with Tyrosine hydroxylase (1∶100, T8700, Sigma) and anti-B220 (1∶200; Clone 6B2 kindly provided by Dr Martijn Nolte, Sanquine, Amsterdam) antibodies. Before primary antibody incubation, sections were post-fixed with cold acetone (2 min) and followed by pretreatment with Na Azide (0.1%) and H_2_O_2_ (0.3%) for 15 min. A 30 min blocking step with BSA 1% was performed prior the incubation with the primary antibodies (1 hr). Anti-rat AF488 (1/400) and anti-rabbit AF546 (1/400) were incubated for 1 and 2 hrs, respectively at room temperature.

### Image acquisition

Preparations were examined by use of an Olympus BX4 epifluorescence microscope (Olympus America, Center Valley, PA). Immunohistochemical labelled tissues were visualized using a Zeiss LSM510 Meta confocal microscope (Cell Imaging Core, KU Leuven). The following lasers and emission filters were used to visualize the labeled structures and collect images: multiline Ar laser at 488 nm (used for the excitation of FITC); emission filter 535±15 nm; 543 nm HeNe laser (used for Cy3); emission filter 575±630 nm; and 633 nm HeNe laser (used for Cy5); emission filter 650±700 nm. The confocal images were collected using the optimal pinhole size for the 63X oil objective or for the 20X air objective and confocal stacks were taken with *z*-axis step of 0.5 µm (63X objective) or 1 µm (20X objective).

## Results

### Tracer application

**Figure 1:**
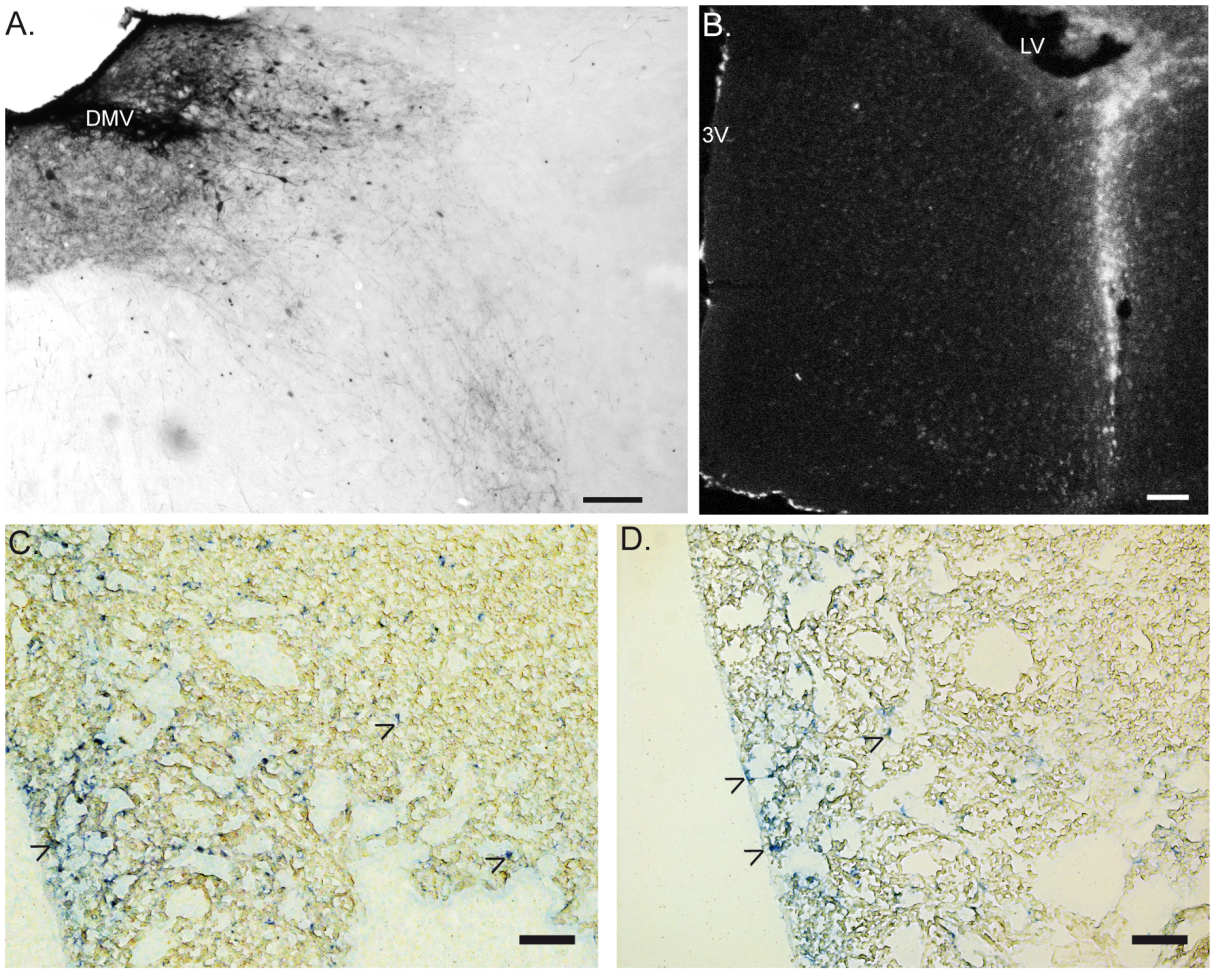
Injection sites of the neuronal tracer and deep cervical lymph nodes. Panel A shows the site of dextran amine injection at the level of the DMV, 19 days after injection. The tracer was revealed by DAB staining. B. Epifluorescent picture shows the distribution of Texas-red dextran amines after lateral ventricle injection. In the panels C & D, arrow heads show the presence of dextran amines (revealed by phosphatase alkaline staining to amplify the Texas red signal of the tracer) in the deep cervical lymph node. Of note, the presence of the tracer was found in all LV injected mice (D) and occasionally in DMV-injected mice (C). 3 V: third ventricle. LV: lateral ventricle. DMV: dorsal motor nucleus of the vagus. Scale bar represents 0.1 mm.

Injection of the tracer in the DMV (Fig. 1A) was achieved in 4 out 9 mice for the biotin-dextran and in 2 out 6 mice for the Texas red-dextran. Two extra mice were injected in the lateral ventricle (LV) to evaluate possible unspecific staining in the peripheral tissue (Fig. 1B). Leakage of the tracer to the cerebrospinal fluid is drained by the deep cervical lymph node (DCLN) and then released in the bloodstream [20]. In line we observed some trace of the dextran amine in the deep cervical lymph node in lateral ventricle injected mice and to a limited extent in some of the DMV injected mice, nineteen days after injection (Fig. 1 C&D).

### Vagal innervation of the spleen

**Figure 2:**
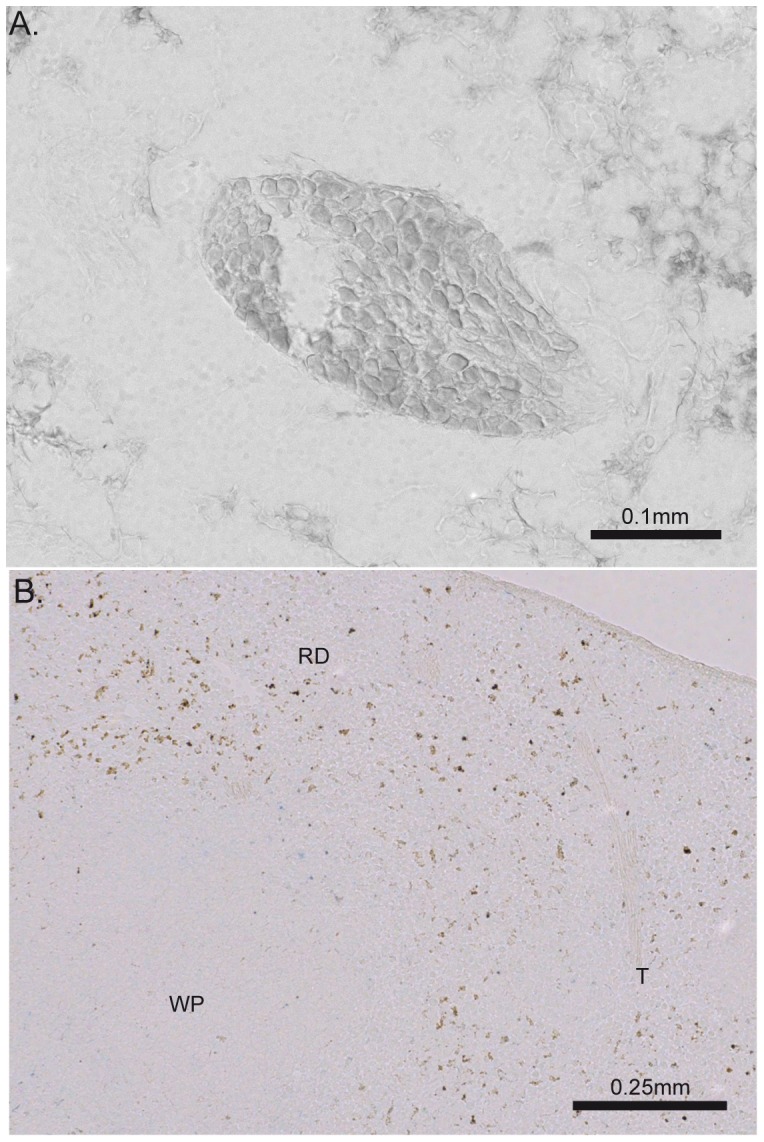
Distribution of dextran-labeled vagal fibers in the mesenteric ganglion and spleen coronal section. No biotin dextran-labeled fibers or terminals were found on coronal section of mesenteric ganglion (A) or spleen (B). Of note, similar observations were obtained with Texas red dextran amine tracer. The brown spots on the spleen section were found in injected and non-injected mice, indicating of a strong endogenous biotin expression. WP: white pulp, RP: red pulp, T: trabeculae.

**Figure 3:**
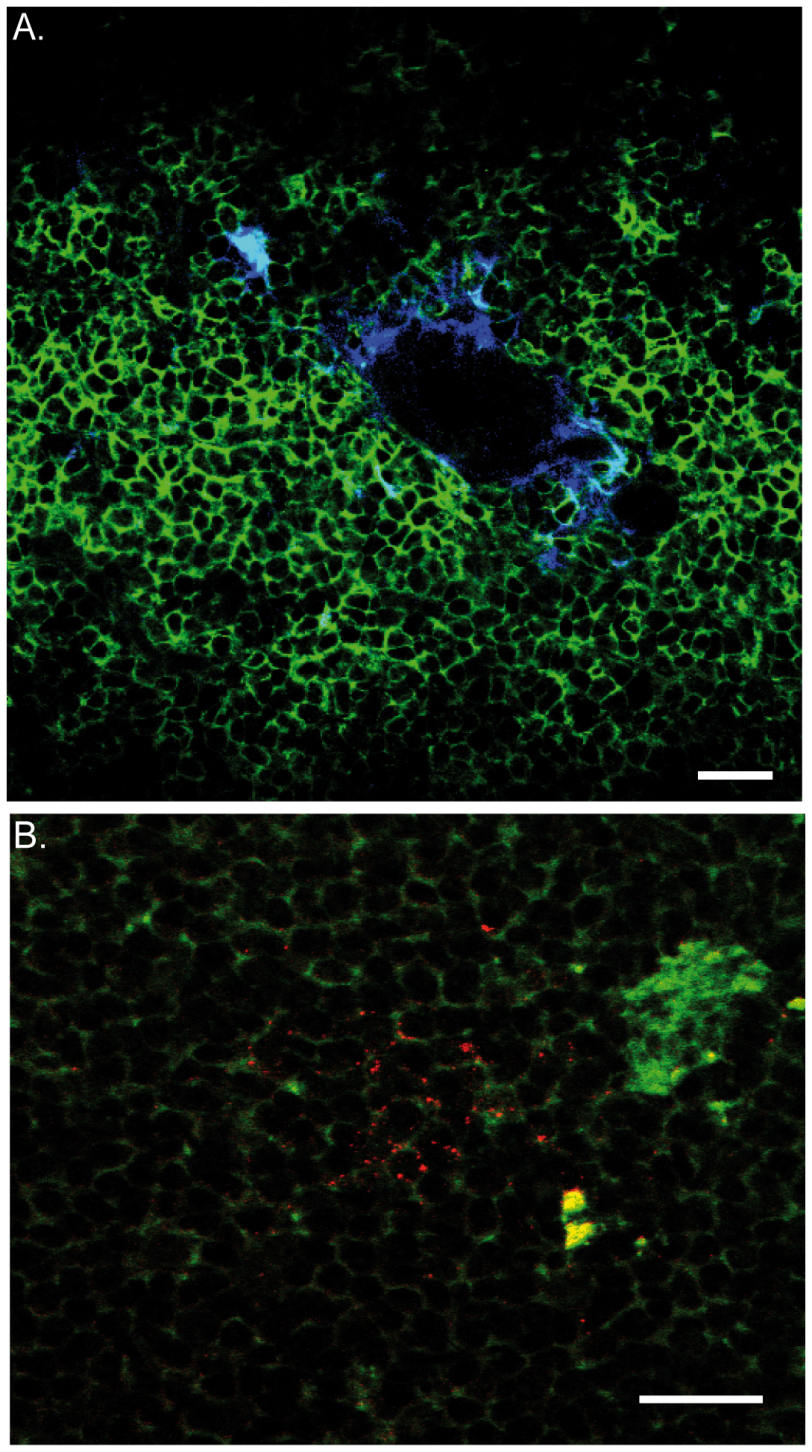
Sympathetic fibers and no vagal innervation of the spleen. A. Tyrosine hydroxylase (TH) staining was used to reveal the sympathetic innervation of the spleen. The central arteriole showed high TH positive fibers (blue) that are in close proximity of the T cells (green). B. Texas red amine signal (red) was found occasionally in the B cells area (green). This dextran amine signal was found in mice that exhibit also dextran amine in deep cervical lymph node, indicating that the spleen signal is the result of tracer leakage into the cerebrospinal fluid. The scale bar represents 20 µm.

The spleen has been proposed to play a central role in the anti-inflammatory effect of the VNS in sepsis [9], [21], [22]. Although previous studies showed neuronal synapses between the vagal efferent fibers and sympathetic cells bodies of the mesenteric ganglia [15], [23], our anterograde tracing experiments did not reveal any dextran positive vagal fibers or terminals in the mesenteric ganglion (Fig. 2A). Similarly, no labeled vagal efferent fibers or terminals were found in the spleen using Biotin-Dextran or Texas-Red conjugated Dextran (Fig. 2B). Only TH positive fibers were found throughout the spleen tissue along the blood vessel ending in the white pulp in close proximity to T cells (Fig. 3A). Occasionally, Texas red dextran was found in the follicular dendritic cells in the B cell area (Fig. 3B) in both DMV and LV injected mice. This non-specific staining was most likely caused by the release of the tracer into the circulation as confirmed by the presence of the dextran in the deep cervical lymph node in both types of injection (Fig. 1 C&D).

### Vagal motor efferent fiber distribution in the intestine

**Figure 4:**
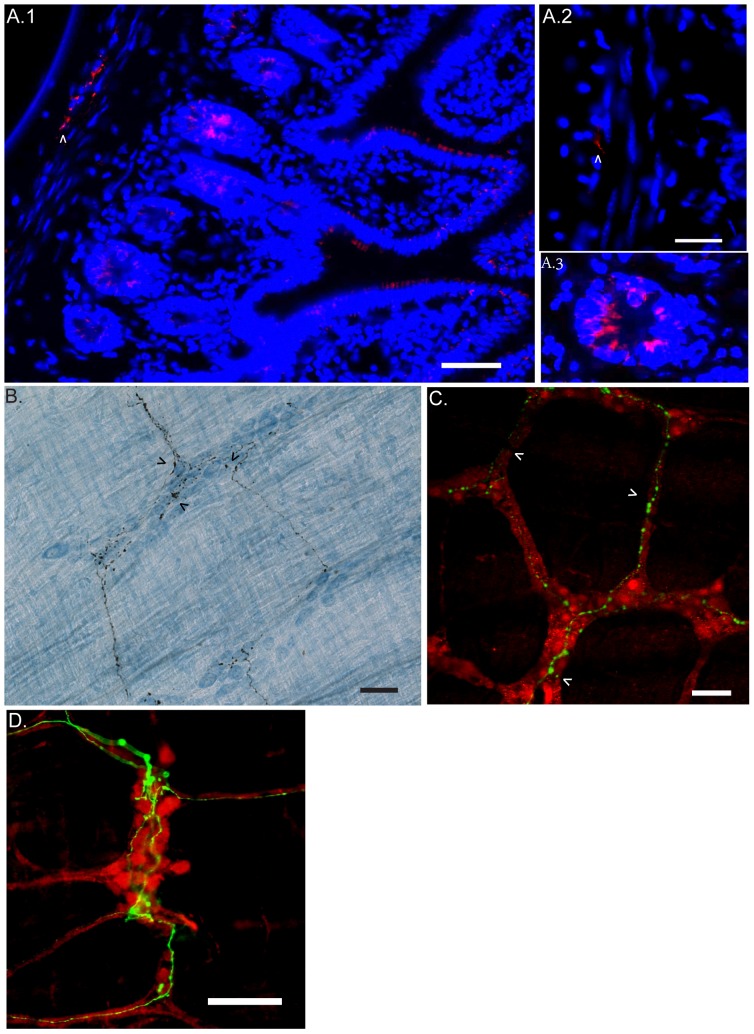
Vagus nerve efferent fibers and terminals reach myenteric plexus region in the intestine. A.1 Vagal motor efferent fibers (red; arrow head) were labeled using fluorescence conjugated streptavidin on 5 µm thin coronal section of small bowel. Labeled vagus fibers were found between circular and longitudinal muscle layers at the level of the myenteric plexus. A.2. High power magnification field showing localization of vagal motor efferent fibers (red, arrow head) between nuclei (blue) of circular (CM) and longitudinal (LM) smooth muscle. A.3. The presence of intrinsic biotin was found in cells of the submucosal crypts. B. Labeled-fibers of the vagal nerve were revealed with ABC/DAB staining protocol while cuprolinic blue was used as pan-neuronal marker to visualize enteric neurons (blue). Labeled vagal efferent fibers and terminals with a basket-like shape terminals were found within myenteric ganglia (arrow head). C. Confocal image showing the presence of the dextran amine in the inter-ganglionic fibers (arrow). D. Epifluorescence image corresponding to vagal efferent fibers (green) densely found at the level of myenteric ganglion (PGP 9.5, red). The scale bar represents 50 µm.

Only DMV-injected mice exhibited dense dextran-labeled vagal fibers in the gut muscularis of the small intestine (Fig. 4). No dextran-labeled fibers were observed in LV injected mice. As previously reported by others [18], we did not find labeled cells bodies in the nodose ganglia 19 days post-injection, confirming the specificity of the labeled fibers to motor neurons arising from the DMV. Dextran amines injection in the DMV of mice provided a similar vagal distribution pattern in the gastrointestinal tract as previously reported in rats using the tracer DiI [14] and with a similar sensitivity and specificity as reported by Walter *et al.* [18].

The distribution of the pre-ganglionic vagal efferent fibers (Fig. 4) was exclusively confined within the myenteric plexus located between the circular and longitudinal muscle layers of the intestine. No labeled fibers or terminal were observed within the submucosal plexus or lamina propria (Figs. 4A.1 & A.2). We did however notice a bright signal in the cells of the submucosal crypts (Fig. 4A.3). Based on the location (inside submucosal crypts) and the shape (round cells), the bright signal is most likely indicative of the presence of endogenous biotin expression in those cell types. In whole mount preparations, biotin dextran amine with nickel enhancement provided a clear distribution of the efferent vagal fibers/terminals connecting to enteric ganglia within the myenteric plexus region. The permanent staining with ABC and cuprolinic blue revealed the morphology of the terminals that synapse with neurons located in the myenteric ganglia (Fig. 4B). Labeling with a secondary fluorescent antibody combined with PGP 9.5 confirmed that vagal fibers and terminals in the small intestine were confined to the myenteric ganglia located at the level of the MYP (Figs. 4C & D).

### Chemical coding of vagal efferent fibers in the small intestine

**Figure 5:**
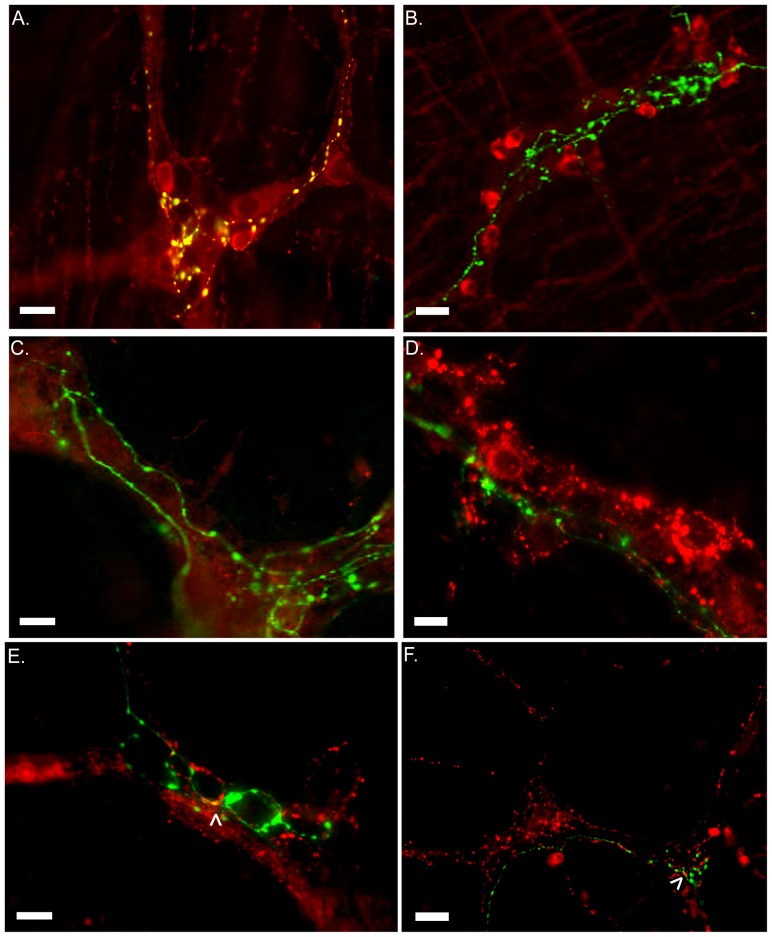
Vagus nerve efferent is fully cholinergic in nature. Epifluorescence images collected for the identification of the neurotransmitters (red) and dextran-labeled vagus efferent fibers and terminals (Green). Choline acetylfransferase (ChAT, A), neuronal nitric oxide synthase (nNOS, B), Tyrosine hydroxylase (TH, C), substance P (SP, D), vasoactive intestinal peptide (VIP, E) and Calcitonin gene related peptide (CGRP, F). Arrow heads point discrete co-localization between Vagus nerve and VIP or CGRP positive structures. Vagus nerve efferent fibers and terminals are only positive for ChAT and located in close proximity to ChAT and nNOS enteric neuronal bodies. Scale bars represent 20 µm.

Immunoreactivity for various neurotransmitters and peptides confirmed that the vagal pre-ganglionic fibers were mainly positive for choline acetyltransferase (ChAT) (Fig. 5A). Dextran-labeled axons/terminals were negative for neuronal Nitric Oxide Synthase (nNOS), Tyrosine Hydroxylase (TH), Substance P (SP), Vasoactive Intestinal Peptide (VIP) and Calcitonin Gene-Related Peptide (CGRP) (Fig. 5B–F).

In rare occasions, we found that efferent vagus nerve terminals in the small intestine show VIP and CGRP immunoreactivity (at occasional points) at the level of the myenteric plexus region (Fig. 5 E&F, arrow head). However with the methodology used here, it is impossible to discriminate between VIP and CGRP stored in vagal nerve terminals or overlap of enteric nerve fibers with vagal efferents (Figs. 5 E&F).

### Contact between enteric neurons and macrophages

**Figure 6:**
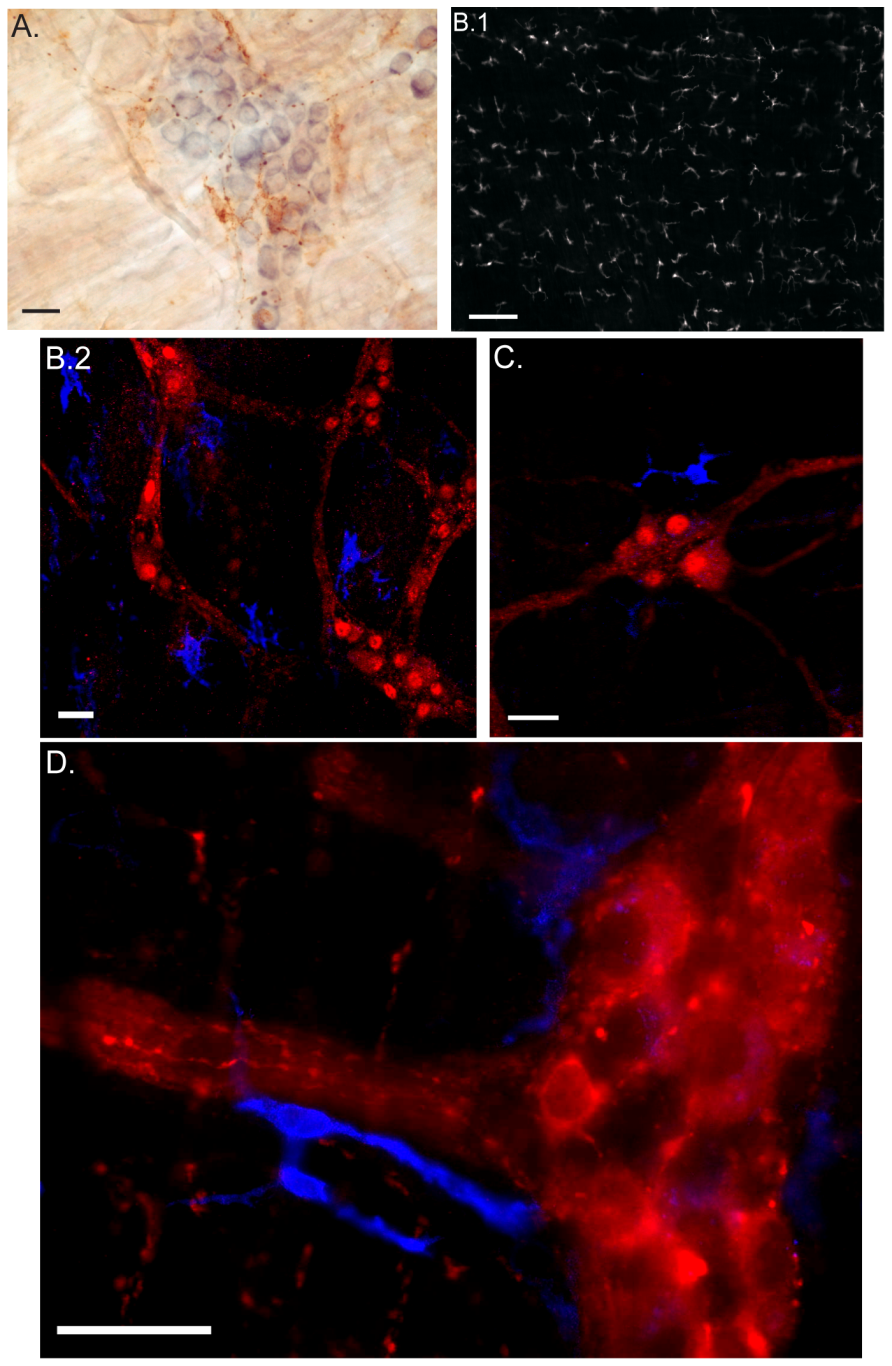
Intestinal resident macrophages are located in proximity to enteric neurons. A. Staining of F4/80 positive intestinal resident macrophages (brown) surrounding a myenteric ganglion (blue). Efferent vagus nerve fibers are shown in black. B.1 Regular distribution of resident macrophages (F4/80) in the muscularis of the murine small bowel. B.2 & C. Confocal image showing the distribution of the resident macrophage (F4/80, blue) close to enteric neurons (PGP9.5, red) in the muscle layers of the small intestine. D. Epifluorescence image showing the presence of resident macrophages (F4/80, blue) in close proximity to ChAT positive enteric ganglion (red). Scale bar represents 25 µm, except for B.1 it represents 0.1 mm.

Intestinal handling is known to activate the resident macrophage network (F4/80^+^CD11b^+^) that resides in the gut muscularis. VNS applied before surgery implies that ACh released from the vagus nerve suppresses the activation of these resident macrophages (F4/80^+^CD11b^+^). Using immunohistochemistry and immunofluorescence techniques we observed a regular distribution of the resident macrophages (F4/80^+^CD11b^+^) located between the longitudinal and circular muscle layers of the small intestinal (Fig. 6) with some of the macrophages located closely to the myenteric ganglia (Figs. 6A&D). The cholinergic dextran amine labeled terminals did not make contact with resident macrophages. We observed only ‘basket-like’ endings around the cell bodies of the myenteric neurons (Fig. 6A). Figure 6B.2 and C show the location of intestinal resident macrophages in relation to PGP 9.5 positive enteric neurons while figure 6.D shows a higher magnification of two intestinal resident macrophages in the proximity of one ChAT positive enteric ganglion. Based on the distribution of the pre-ganglionic vagal fibers, we propose that the dampening effect of VNS on macrophage activity is mediated through vagal interaction with postganglionic neurons (enteric neurons).

### Chemical coding of the myenteric neurons targeted by the vagus nerve

**Figure 7:**
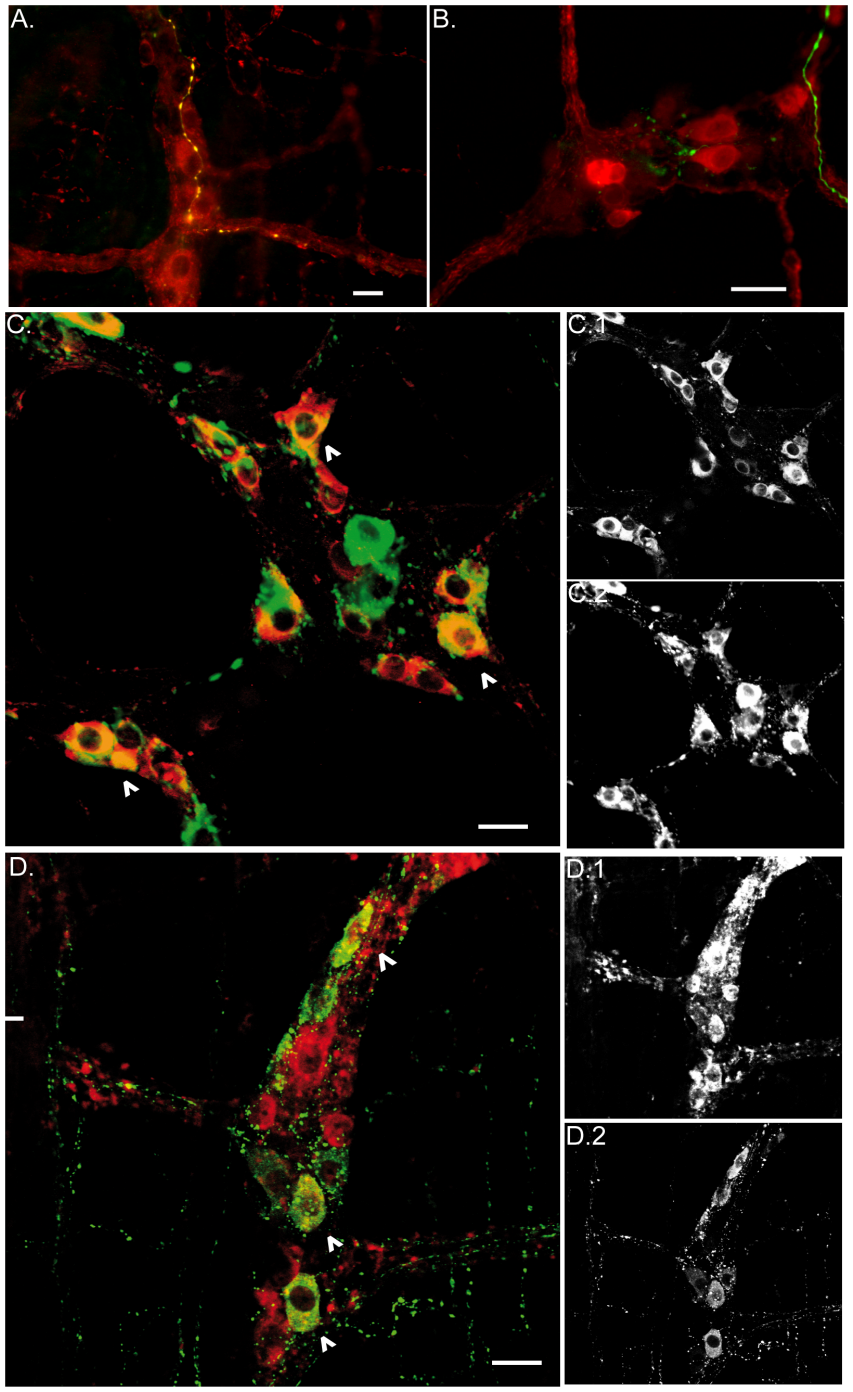
Vagus nerve efferent fibers and terminals are close to cholinergic and nitrergic enteric neurons. A. Epifluorescence image shows dextran-labeled vagal efferents (green) that co-localize with ChaT (yellow), and are in close contact with ChaT positive enteric neurons (red). B. Epifluorescence image shows labeled vagal efferent fibers (green) making contact with nNOS positive neurons (red). Of note, cholinergic neurons, and to lesser extent nitrergic neurons, are the main population targeted by the vagal efferent fibers. C. Confocal image of VIP (red) and nNOS (green) myenteric neurons. Most of the cells bodies exhibit co-localization of these two neurotransmitters (arrow head). D. Confocal image of VIP (red) and ChaT (green) myenteric neurons. Occasionally myenteric neurons showed immunoreactivity for both neurotransmitters (arrow head). C1 and C2 show the distribution for the nNOS and VIP positive cells bodies, respectively. D1 and D2 show the distribution for ChaT and VIP positive cells bodies. Scale bar represents 20 µm.

The vagal efferent terminals were found mainly close to nNOS and ChAT positive myenteric neurons (Fig. 7A & B). nNOS immunoreactive cells bodies showed an extensive co-localization with VIP (Fig. 7C) while only a few ChAT positive neurons exhibited VIP immunoreactivity (Fig. 7D).

**Figure 8:**
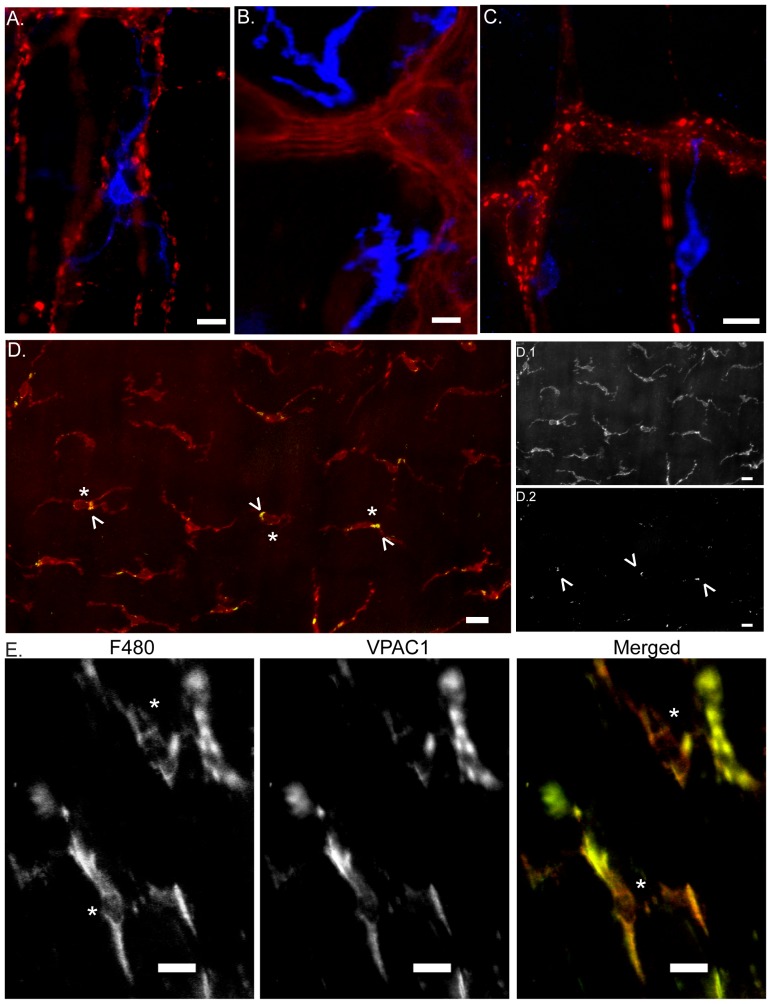
Neuronal fibers and intestinal resident macrophages: neurotransmitter and receptor expression. Epifluorescence images show F4/80 positive intestinal resident macrophages (blue) located in close proximity to inter-ganglionic enteric fibers positive for ChAT (A), nNOS (B) and VIP (C). D. Intestinal resident macrophages (F4/80, red, D.1) expressing α7 nicotinic receptor (green, arrow heads, D.2). E. Resident macrophages of the gut muscularis (F4/80, red) express VPAC1 receptors (green). Asterisk corresponds to intestinal resident macrophages located at the level of the myenteric plexus region. The other macrophage is located at the level of the submucosal plexus. Scale bar represents 10 µm from A–C and 20 µm from D–E.

Double labeling procedures were performed to identify the neurochemical phenotype of the enteric fibers running close to the macrophages at point far from the myenteric ganglia. So VIP, ChAT and nNOS immunoreactive fibers were all found in close proximity to F4/80 positive macrophages (Fig. 8A–C).

Although two commercial antibodies for α7 nAChR have been successfully used to label immune cells in the rat gut or murine airway epithelium [24], [25], we failed to observe specific labeling in whole mount preparations of the small intestine. The antibodies used provided the same signal in non primary controls specimens and also in tissue from α7 nAChR^-/-^ mice (data not shown). In contrast, the fluorescent conjugate of the nicotinic receptor antagonist bungarotoxin specifically stained the resident macrophages present in the gut muscularis (Fig. 8D). No specific staining was detected on muscle layers collected from α7 nAChR^-/-^ mice (data not shown). Interestingly, these resident macrophages also showed immunoreactivity for the VIP receptor VPAC1 (Fig. 8E), suggesting that VIP could participate in the anti-inflammatory effect of vagus nerve stimulation.

## Discussion

In the present study, we show that the vagus nerve does not directly interact with resident macrophages in the intestine or spleen. In the intestine, vagal efferent fibers interact with nNOS and ChAT positive myenteric neurons, with nerve endings in close proximity to resident macrophages carrying α7 nAChR. Of note, nNOS and some ChAT positive neurons co-expressed VIP, while VIP positive nerve fibers were identified in the vicinity of VPAC1 positive macrophages, suggesting that VIP could also be involved in the immuno-modulatory effect of VNS. In contrast, no evidence indicating vagal innervation, either direct or via the mesenteric ganglion was obtained for the spleen. Based on these data, we conclude that vagal modulation of the intestinal resident macrophages is indirect, most likely through cholinergic and nitrergic/VIPergic enteric neurons.

Electrical stimulation of the vagus nerve, before and after intestinal manipulation, prevents the inflammatory response triggered by intestinal handling and consequently reducing postoperative ileus [12]. This effect results from the inhibition of the resident macrophages through acetylcholine-mediated activation of nicotinic receptors. To what extent the vagus nerve directly interacts with these resident macrophages however has not been studied. Using anterograde tracing, we aimed to detect the efferent nerve fibers innervating the intestine and spleen in a mouse model. Dextran amine was the most suitable tracer due to its low toxicity (water solubility) allowing multiple injections with minimal impact on the general condition of the mice. The duration of the transport of the tracer from the source (cell body) to the terminals relies on the type of neuron and its activity. In our case, the time period of 19 days was required for the motor axonal fibers to efficiently transport the tracer to the terminals. Following this time period, dextran amine selectively labeled the motor neurons axons. Indeed, no dextran-labeled cells bodies were found in the nodose ganglia while no labeled fibers were found in the gastro-intestinal tract when the injections were outside the DMV (data not shown).

Using this technique, dextran-labeled vagal efferent fibers were densely found in the small intestine at the level of the myenteric plexus located between the longitudinal and circular smooth muscle layers, but not in the submucosal compartment. In the myenteric plexus region, the vagal efferents endings were predominantly found around ChAT immunoreactive cells bodies, but could not be detected in the vicinity of resident macrophages. Instead, resident macrophages were found in close proximity to cholinergic fibers, i.e. mainly inter-ganglionic nerve fibers. Especially as we found no cholinergic vagal efferents in the vicinity of the macrophages, our data strongly suggest that mainly cholinergic enteric neurons rather than vagal nerve fibers directly interact with the resident macrophages. In addition to cholinergic neurons, we also observed close contacts between vagal efferents and nNOS positive enteric neurons. Similar to cholinergic nerve fibers, nNOS positive nerve fibers were found in close proximity to resident macrophages, suggesting a potential role modulating macrophage function. Taken together, our data indicate that the vagus nerve does not directly interact with the resident macrophages, but most likely modulates these immune cells through cholinergic and to a lesser extent nitrergic enteric neurons.

Vagus nerve stimulation potently suppresses the inflammatory response in sepsis and improves survival. This effect has been proposed to be mediated by vagal activation of sympathetic neurons in the coeliac ganglion innervating the spleen [9], [21]. Although dextran amine is a sensitive anterograde tracer to label complex brain circuits [26], [27], we were unable to detect this anterograde tracer in the mesenteric ganglion or in the spleen, indicating that no vagal fibers arising from the DMV are projecting to the spleen. The lack of anterograde tracer in the spleen coincides with data recently published [28]. Using transgenic GFP-ChAT mice to visualize pre and postganglionic cholinegic neurons, only a sparse ChaT positive innervation was shown in the spleen consisting of neuronal fibers of spinal origin (sympathetic) around arterioles and in lymphocyte-containing areas of the white pulp. The absence of labeled fibers in the celiac-superior mesenteric ganglia found in our current study did not correlate with previous studies using DiI anterograde tracer [15], [16]. The labeling period (19 days) for dextran amines may not be sufficient to reveal the moderate density of the vagal fibers in the ganglia previously reported [18] even though it successfully labeled the vagal efferent fiber throughout the entire gastrointestinal tract.

In the intestine, the neurons contacted by the vagus nerve are predominantly cholinergic. In the spleen, acetylcholine released by T cells is proposed to suppress splenic macrophages [10], [28], most likely though the activation of the α7 nicotinic acetylcholine (α7 nAChR) receptors [1]. In the intestine, we collected evidence that this receptor is located on resident macrophages and mediates the anti-inflammatory effect of vagus nerve stimulation in a model of postoperative ileus ([29] in press). In the present study, we confirm these data by immunohistochemistry. The use of the two commercially available antibodies for α7 nAChR provides a similar staining pattern of α7 nAChR on muscle layers as previously reported [30], [31]. However, these antibodies exhibit similar results in α7 knock out mice, indicating that these antibodies lack specificity for α7 nAChR. In contrast, using bungarotoxin staining, we indeed revealed the presence of α7 nAChR (only) on resident macrophages. Interestingly, we also demonstrated close interaction between vagal efferents and nitrergic neurons co-expressing VIP. These NO/VIP positive neuronal fibers were found in close proximity to resident macrophages that express VPAC1 receptors suggesting that not only ACh, but also VIP and NO may modulate the function of resident intestinal macrophages [32], [33].

In summary, no evidence supporting vagal or cholinergic innervation of the spleen could be provided. However, we collected neuro-anatomical evidence that the vagal modulation of intestinal resident macrophages is indirect and mainly involves cholinergic enteric neurons. Based on these data, we speculate that the cholinergic anti-inflammatory input to the intestine is mediated and thereby amplified by the enteric nervous system.
